# Report on the Pathology and Treatment of Epidemic Cholera

**Published:** 1868-09

**Authors:** N. S. Davis

**Affiliations:** Professor of Practical and Clinical Medicine, in Chicago Medical College


ARTICLE XXXI.

### REPORT ON THE PATHOLOGY AND TREATMENT OF EPIDEMIC CHOLERA.

BY N. S. DAVIS, M.D., Professor of Practical and Clinical Medicine, in Chicago Medical College.

Presented to the Illinois State Medical Society, May, 1868.

At the annual meeting of the Illinois State Medical Society for 1867, the writer was appointed a Special Committee to report at this meeting on the causes, pathology, and *treatment* of that much dreaded scourge called, variously, Epidemic, Spasmodic, and Asiatic Cholera. But few subjects could be selected from the whole field of Therapeutics and Practical Medicine concerning which a greater diversity of views have been presented and acted upon, or concerning which the professional mind is more unsettled, than the one now under consideration. In the following report no attempt will be made to criticise the views of others, or to reconcile the apparently conflicting statements found in our medical literature. On the contrary, it will be limited mostly to a plain statement of the writer's own views of the treatment of cholera, as derived from direct observation at the bedside of cholera patients.

My opportunities for observation commenced in the summer of 1849, during the severe epidemic which prevailed at that time in the city of New York, and were continued in Chicago during the epidemics of 1850–51–52–54 and '66. To occupy the whole field of investigation assigned to me by the Society would require the writing of a volume rather than a report suitable for reading to a Society like this. And, inasmuch as the causes of cholera are still a subject of active investigation, they will be alluded to simply in this paper.

The essential cause of cholera, or the specific cholera poison (if such an agent exists), is still involved in mystery. The most popular theory at the present time is, that it consists of a highly poisonous organic body, developed in the gastric and intestinal evacuations of patients laboring under cholera, and capable of being transported from place to place by such evacuations, either freshly voided, or by adhering to clothing, or by diffusion in the soil (Pettenhoffer), when there is a water-bearing stratum near the surface. Some observers have claimed to discover the specific poison in the cholera dejections, by the aid of the microscope. That such dejections contain an abundance of animalcules when voided, and that an abundant crop of vegetable fungi or sporules appear in the same discharges when allowed to stand from two to five days in a temperature above 60° Fahrenheit, is unquestionably true.

I have made quite a number of microscopic examinations of the discharges from the bowels, both from patients laboring under ordinary serous diarrhœa and epidemic cholera. In all of them, when examined soon after having been voided, animalcules were abundant, especially those called *vibriones;* and in the samples taken from two well-marked cases of cholera, besides the ordinary vibriones, there appeared two or three animalcules of larger size, more caudate, and moving across the field with great activity. After the discharges had been allowed to stand from two to five days, fungoid growths appeared in abundance, that were not present when first voided.

These are not presented as specimens of the specific cholera poison. On the contrary, so far as my examinations have been extended, they lead rather to the conclusion that these products may be found in all serous discharges from the alimentary mucous membrane, whether in cholera, cholera morbus, or simple diarrhœa. But if the untiring scientific researches of the profession have not yet fully identified some *materies morbi* as the specific cause and propagation of epidemic cholera, they have done what is of equal or greater practical value, namely, pointed out the meteorological and sanitary conditions favorable to its development and fatality. Meteorologically, these conditions are, a warm, damp, still atmosphere, with deficiency in free electricity and ozone. But with these must coëxist such local sanitary conditions as are capable of furnishing either animal or vegetable miasms, or both combined. Hence the disease has always been most prevalent and fatal where human beings were most crowded together, with the least attention to ventilation and cleanliness, whether in caravans of pilgrims, in emigrant ships, or the low and uncleanly parts of cities. And, whenever invading country districts, it has chosen the alluvial valleys and malarial districts, and wholly passed by the more rugged and primitive geological districts. The truth and value of these observations are both illustrated by a close study of the rise, progress, and decline of an epidemic of cholera in any of our populous cities. For instance, here are two maps, one of Chicago and the other of New York, on which are indicated the districts most infected with cholera in the summer of 1866.

| | | | | | |
|---|---|---|---|---|---|
| 1866. In 7th Ward, | 1 case to | 100 population. |
| 14th " | 1 " | 95 " |
| 1st, 2d, 8th, 9th " | 1 " | 250 " |

$\frac{1}{3}$ of whole number of cases in 7th, 14th, and 16th Wards.

1867. August and September.

| | | | |
|---|---|---|---|
| 1st, 2d, 3d, 9th, 10th Wards, | 63,000, | 1 death in | 335. |
| 5th, 6th, 7th, 13th, 14th " | 69,000, | 1 " | 125. |

*Pathology.*—My views of the pathology of cholera can be stated in a few words, as follows:

A direct diminution of that elementary property of living, organized matter, which I call vital affinity, coupled with impaired contractility, or partial paralysis of the capillary vascular system, or, more properly, of the arterioles, and special morbid sensibility of the mucous membrane of the alimentary canal. The coëxistence of these pathological conditions causes, first, diminution of organic changes, and consequent diminution of secretion and calorification, with a sense of weakness and lassitude, accompanied by pallor, intestinal commotion, and occasional thin stools, constituting what has been called cholerine, or the first stage of epidemic cholera. Second: Still further diminution of secretion and calorification, with complete relaxation, or paralysis of the arterioles, which, coöperating with the morbid sensibility of the mucous tissues, causes a rapid exudation or exosmose of the serous and saline elements of the blood from their capillaries, and their rapid ejection by vomiting and purging, constituting the second or active stage of the disease. Third: The entire suspension of organic changes, including secretion and calorification, with such exhaustion of the watery and saline elements of the blood, as leaves the whole mass viscid and unoxygenated, constituting the stage of collapse.

After much clinical study and observation, during repeated epidemics of cholera, it has seemed to me that the foregoing essential pathological conditions most fully explain both the symptoms during life, and the *post mortem* appearance after death. Whether they arise from the introduction into the blood of some specific *materies morbi* capable of propagation and transportation, or from the influence of some special combination of atmospheric and sanitary conditions, remains for future investigations to determine.

*Treatment.*—The pathological conditions stated, not only afford a satisfactory explanation of the symptoms and results of cholera, but they equally afford clear indications for a rational mode of treatment. These indications are: 1st. To increase vital affinity and vascular contractility, and diminish the morbid sensibility of the stomach and bowels, thereby preventing the development of the second stage of the disease. 2d. To add to the first indication such measures as will most speedily arrest the rapidly exhausting discharges that characterize the second stage; and 3d. To add to both the foregoing such means as will be most efficient in supplying the lost elements of the blood, renewing the sensibility of that portion of the organic nervous system called vaso-motor, and promoting the decarbonization of the blood. If we were certain that the disease was produced by the introduction into the system of some specific *materies morbi*, of course there would be an indication for treatment, of prior and paramount importance to all those just enumerated, namely, to administer some remedy which would be capable of directly neutralizing or expelling the specific poison. But as we neither know the nature of the supposed poison nor its antidotes, we must pass this item by for the present, and confine our attention to the consideration of such means as we deem best calculated to fulfil the several indications previously stated.

In doing this, we shall adhere rigidly to the purpose announced at the commencement of this article. To fulfil the indications presented in the first stage of cholera, three things are of paramount importance, *viz.:* rest, pure air, and such medicine as will produce a decided tonic and mildly anodyne influence. By tonics here I do not mean nervous exhilarants, or what are popularly styled diffusible stimulants, but such medicines as produce an increase of vital affinity and consequent tonicity of the tissues. I require the patient to maintain the recumbent position, restrict his diet mostly to milk porridge (boiled milk and wheaten flour), ventilate the room freely, and if the air is damp and impure, increase the ozone by either sprinkling a solution of permanganate of potash or allowing the slow oxidation of phosphorus in it, and give some one of the mineral acids, with a small proportion of an opiate. A formula that I use more frequently than any other is as follows:

℞. Aromat. Sulph. Acid, ---- ------------ ʒiij.

Sulph. Magnesia, -------------------- ʒiij.

Tinct. Opii, ------------------------ ʒiij.

Aqua Menth., ---------------------- ℥iij.

Mix, and give a teaspoonful, in sweetened water, every two, three or four hours, according to the severity of the symptoms, until all intestinal commotion ceases, and the evacuations become consistent and natural. A dozen combinations might be named, all calculated to produce similar results, but it would be a waste of your time.

In the second stage, I require the same attention to the atmosphere of the room, and the same position of the patient, with the addition of dry warmth to the extremities and strong mustard sinapisms over the abdomen and nearly the whole length of the spine. But now the whole mucous surface, instead of continuing its natural function of *absorption*, has reversed its action, and is rapidly discharging the serum and salts of the blood, with more or less detachment of its own epithelium, as shown by the copious and frequent discharges up and down.

To arrest this process I rely mainly on the following:

℞. Sulph. Morph.,------------------------ 3grs.
Calomel, ------------------------------12grs.
White Sugar,--------------------------- ℨj.

Mix, and divide into 12 powders, one of which is given *immediately* after every turn of vomiting, whether it be once in five or forty minutes. The craving for drink is satisfied as much as possible by small pieces of ice. After every evacuation from the bowels, I direct to be thrown into the rectum an enema of two ounces of cold water, containing half a grain of sulphate morphia, and 10 grains of acetate of lead. If the cramps are very severe, a moderate dose of morphia may be injected hypodermically with advantage. But sufficient caution should be used to avoid *narcotizing* the patient. In using the powders and enemas just mentioned, it is of very great importance to note the manner of their administration. They should be given *immediately after* every evacuation. The act of vomiting is one that cannot be performed continuously; and medicine swallowed just as soon as the effort ceases, and the patient inclines to turn his head down to rest, will remain in contact with the mucous membrane long enough to make some impression before the muscular structures have regained the power to renew the act of vomiting.

But if the physician yield to the inclinations of the patient and nurse to let him rest a *few minutes*, or, as they often express it, "to let his stomach get settled a little," a fresh supply of serous fluid will have accumulated in the stomach, and the muscular fibres are again ready to act, and now the medicine is no sooner swallowed than it is returned by vomiting, and no advantage is gained. The rule to which I call your attention so strongly is applicable to the administration of all medicines or restoratives during the active stage of vomiting. When the vomiting is so far checked as to admit of half or three-quarters of an hour between the paroxysms, I begin to administer restoratives in the form of animal broth, well salted either with common salt or chlorate potassa, and strong coffee; the first to nourish and supply the lost elements of the blood, and the second, to sustain the wakefulness and activity of the nervous centres. They must be given at first in only dessert-spoonful doses, but may be increased, if found to be retained by the stomach. If the active progress of the disease is arrested during the early part of the second stage, before great exhaustion has been induced, the remainder of the treatment may be such as we have indicated as applicable to the first stage. But if the discharges have continued until the surface is much shrunken, the pulse very feeble, the urine and other secretions suspended, so soon as the discharges are slightly checked we must not only resort to the cautious but diligent use of the restoratives already mentioned, but remedies to sustain the sensibility of the vaso-nervous system, and consequently secretion, must be resorted to. Of these, probably strychnia is the most reliable. A sugar-coated granule, containing $\frac{1}{32}$ of a grain, may be given every half hour or hour, according to the urgency of symptoms, until some effect is induced. Or, if the stomach will retain it, a teaspoonful of the following formula may be given, in a tablespoonful of water, just as often, *viz.:*

℞. Tinct. Cinnamon, ---------------------- ℥j.

Nitrous Ether, ---------------------- ℥ij.

Tinct. Nux Vom., ------------------ ℥j. Mix.

This has the advantage of being more diuretic, but its greater bulk and pungency renders it more liable to be rejected. If the patient passes into full collapse, the judicious administration of dilute mucilaginous solutions of non-purgative salts, milk, animal broth, and strychnia, with a continuance of dry warmth externally and free ventilation, will afford the best chance of recovery; but the majority of patients in this stage prove insensible to any and every kind of treatment. If the cholera attack is arrested at any stage, and the patient passes into a slow consecutive fever, it must be treated on the same principles as the ordinary enteric typhoid fever. I have thus endeavored to give you, in the shortest possible space, the principles which govern my treatment of epidemic cholera, and some of the medicines and formulæ on which I most rely.

I have used a great variety of remedies in the second or active stage of this disease. I have seen a few cases promptly arrested by the internal use of chloroform, in doses of 10 drops every 20 minutes, and a larger number by the combination of chloroform, Cannabis Indica and morphine, called *chlorodyne.* I have treated some with salines—some with salt and mustard emetics—have bled some from the arm, and cupped others both over the spine and epigastrium; and have seen several treated with purgatives, mercurial, saline, and oleaginous. And I have seen some patients recover under all these kinds of treatment, and have seen a larger number recover under no treatment at all, except rest and diluent drinks. The special mode of managing cholera recommended in this paper I was led to adopt during the epidemics of 1849 and 1850, and through all my subsequent experience it has been with me far more successful than any other plan that I have tried, or seen tried by others.

I think the most *fatal* of all the methods of treating cholera that have yet been practised is that which relies mainly on opiates and alcoholic inebriants. In conclusion, I would make one suggestion. It is now very generally considered that sulphate of iron and permanganate of potassa are the most reliable disinfectants for rendering innoxious the dejections of cholera. If there is any truth in the theory that cholera is caused by a specific poison, derived from cholera dejections, would not the early and persistent internal administration of sulphate of iron neutralize the poison in the system, and arrest the disease? Has it or the sulphites been tried?

A few pages might be added on paphylaxis, but I have already trespassed on your patience too far.

